# Warning signals of biodiversity collapse across gradients of tropical forest loss

**DOI:** 10.1038/s41598-018-19985-9

**Published:** 2018-01-26

**Authors:** Fabio de Oliveira Roque, Jorge F. S. Menezes, Tobin Northfield, Jose Manuel Ochoa-Quintero, Mason J. Campbell, William F. Laurance

**Affiliations:** 1 Centre for Tropical Environmental and Sustainability Science (TESS) and College of Science and Engineering, James Cook University, Cairns, QLD 4878 Australia; 2 Universidade Federal de Mato Grosso do Sul, Campo Grande, MS Brazil; 3 Marco and Louise Mitrani Department of Desert Ecology, The Swiss Institute for Dryland Environmental & Energy Research, Ben Gurion University of the Negev, Beer-Sheva, Israel; 4 Instituto de Investigación de Recursos Biológicos Alexander von Humboldt, Bogotá, Colombia

## Abstract

We evaluate potential warning signals that may aid in identifying the proximity of ecological communities to biodiversity thresholds from habitat loss—often termed “tipping points”—in tropical forests. We used datasets from studies of Neotropical mammal, frog, bird, and insect communities. Our findings provide only limited evidence that an increase in the variance (heteroskedasticity) of biodiversity-related parameters can provide a general warning signal of impending threshold changes in communities, as forest loss increases. However, such an apparent effect was evident for amphibians in the Brazilian Atlantic Forest and Amazonian mammal and bird communities, suggesting that impending changes in some species assemblages might be predictable. We consider the potential of such warning signs to help forecast drastic changes in biodiversity.

## Introduction

Our scientific capability to predict abrupt changes in nature—such as from disease epidemics, earthquakes, and insect-plagues—is limited, and this can have serious consequences for human wellbeing^1^. The ecological threshold concept is generally defined as a point where even small changes in environmental conditions will lead to large changes in the state of a system—frequently termed a “tipping point”^2–4^. This concept is useful for guiding decisions made on relatively short timescales and in human-impacted systems. Further, the concept is particularly useful for setting regulatory limits and defining conservation actions involving societal choices and negotiation of values and aims^2,5^.

Changes in environmental conditions can lead to a drastic, nonlinear loss of biodiversity at a threshold of vegetation loss. This could arise from several underlying mechanisms^5–8^. For example, thresholds may originate from species having synchronous responses to habitat loss and fragmentation, such as shrinking forest patch size and increasing isolation, exacerbating Allee effects or breakdowns in mutualisms^6,7,9^. These nonlinear effects of habitat loss and fragmentation on population abundances can cause dramatic shifts in community composition when disturbances surpass a level that the community can no longer tolerate. Another potential mechanism that may affect populations and communities is habitat change feedbacks and cascade effects. For instance, fragmentation may degrade the canopy structure of tropical forests, increasing light penetration, leading to grass recruitment^10,11^. Increased grass abundance can then facilitate fires that subsequently kill fire-sensitive forest trees, causing a feedback loop and abrupt community changes^12^.

Because of the expected consequences of rapid biodiversity loss and the mechanisms exacerbating those consequences, the detection of biodiversity thresholds along forest loss and landscape modification gradients has considerable value for science and conservation. Threshold identification is especially pertinent given the recent catastrophic declines in wilderness areas around the world, particularly those in tropical forests^13^. Detecting warning signals before biodiversity thresholds are surpassed could give conservationists time to act, thereby potentially lessening the extent of negative impacts on biodiversity.

Here, we evaluate potential warning signals that may aid in identifying community proximity to biodiversity thresholds as a response to forest cover loss and fragmentation in the Neotropics. Our identification of threshold proximity is based on the general theoretical idea that a broad class of systems shows “critical slowing down” when nearing a threshold (i.e., systems become slower in recovering from small perturbations which may result in a great variability of trajectories in biodiversity responses among fragments)^3,14,15^. Moreover, deforestation may decrease population sizes and increase the influence of stochastic processes. This may in turn lead to an increase in beta diversity among fragments as forest loss increases^16,17^. As such, we might detect anomalies, such as increased autocorrelation and variance, in biodiversity-related metric values prior to reaching thresholds in response to increasing forest loss^15,18^.

**Figure 1:**
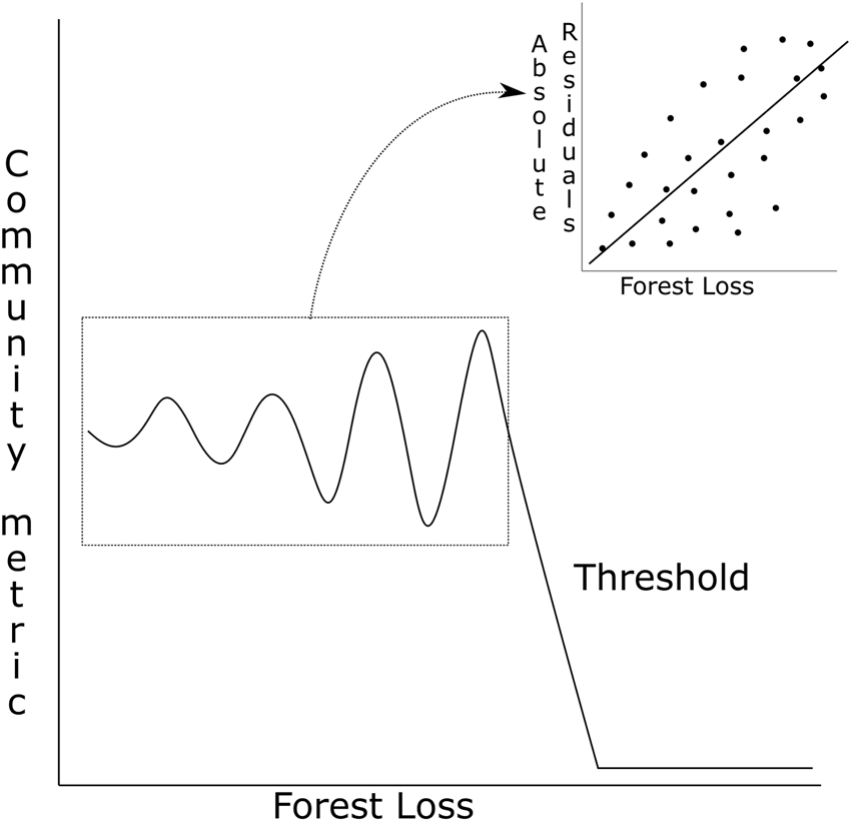
Hypothetical path towards a biodiversity threshold as forest loss increases including increasing heteroskedasticity as a potential warning signal of biodiversity before the threshold.

We focused on heteroskedasticity of biodiversity measures as a potential warning signal of collapse (Fig. 1), because heteroskedasticity is related to greater variance in a system and is simple to calculate and extract from all measures of diversity, including richness and incidence. Heteroskedasticity of biodiversity measures is also one of the better indicators of warning-signals as it is known to minimize false-positive signals due to the inclusion of thresholds, and its use has been validated across a variety of ecological systems^18^. In addition, we explore the possibilities and challenges of using warning signals and threshold information in the context of monitoring different animal groups along gradients of tropical forest loss.

We compiled data from recent Neotropical research papers that documented thresholds for mammal, frog, bird, and insect communities due to forest loss in a variety of forest types and metrics^5,19–21^. We recalculated the threshold values obtained by the original studies in a standardized way using piecewise regression analyses and we focused on identifying trends in heteroskedasticity before these thresholds were reached. We then fitted linear mixed models and linear models to these data and estimated the heteroskedasticity for the subset of data before the thresholds. We selected the residual values before the thresholds to avoid the effect of the abrupt change in the calculation of heteroskedasticity (see Methods).

## Results

**Figure 2:**
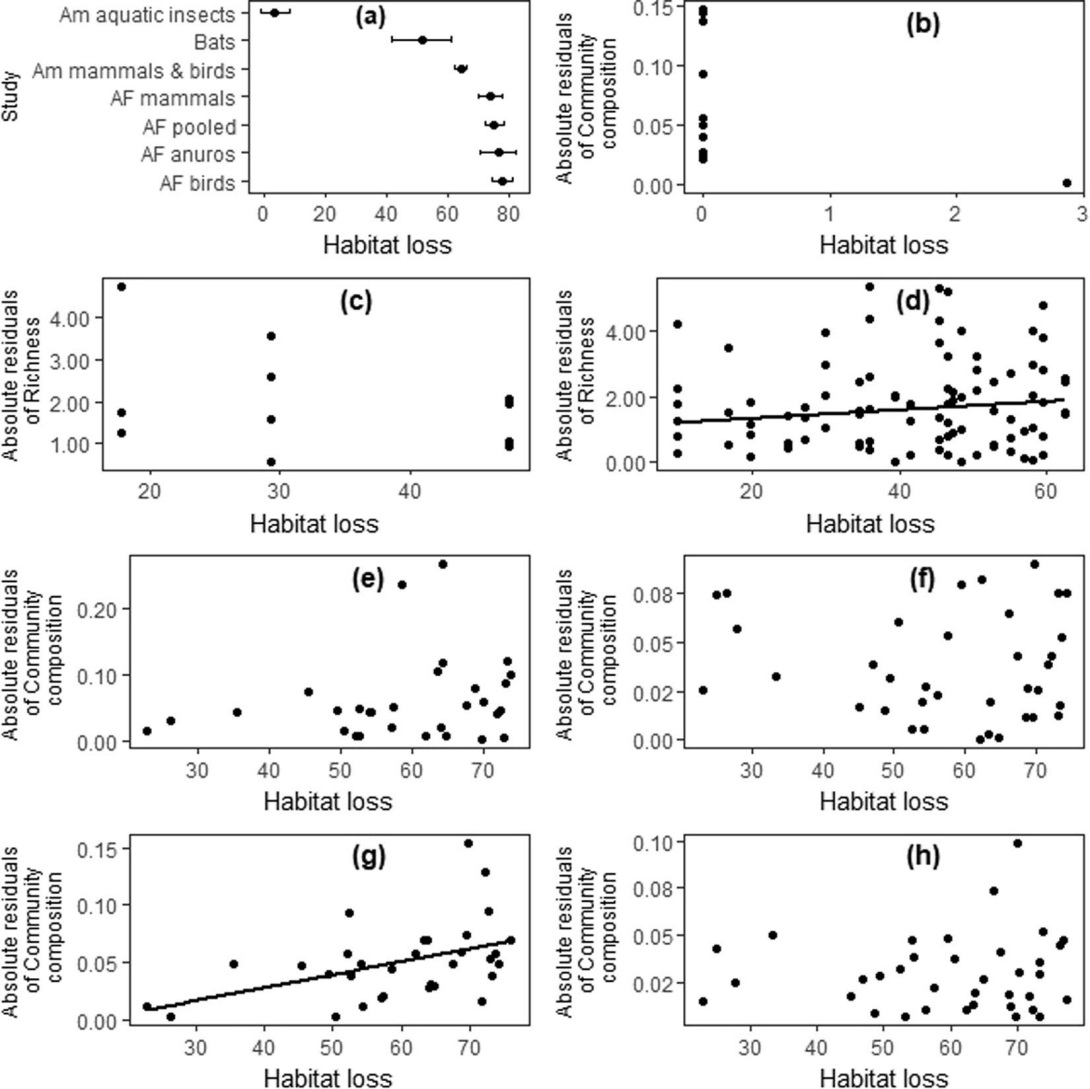
Absolute residuals of regressions of biodiversity metrics and forest loss. According to our hypothesis as deforestation progresses, diversity should increasingly fluctuate until it reaches a threshold point where biodiversity suffers a major collapse. Consequently, absolute residuals in a model of diversity, when examined per unit forest loss, should increase close to the threshold. (**a**) Thresholds detected for different groups along a gradient of habitat loss in the Neotropical region; Absolute residual value for the subset of data before the thresholds for (**b**) Amazonian aquatic insects^21^; (**c**) Cerrado-forest bats^20^; (**d**) Amazonian mammals and birds^19^; (**e**) Atlantic Forest mammals^5^; (**f**) Atlantic Forest pooled groups^5^; (**g**) Atlantic Forest amphibians^5^; and (**h**) Atlantic Forest birds^5^. Black lines show significant relationships (P < 0: 05;F-test) (see Methods). AF – Atlantic Forest; Am – Amazonian.

All piecewise regression models converge to a threshold value. However, some were different from the value reported by the authors of the original studies. More precisely, the threshold values provided by authors for Brazilian Atlantic forest birds, and Amazonian mammals and birds were higher than the confidence interval of our thresholds (Af: Authors: 70.61% of habitat loss, our study: 77.58% ± 3.61 s.e.; Am mammals and birds: Authors: 51.58%, our study: 64.17 ± 2.13; Fig. 2a). Although the significance of these differences was not assessed, the estimated standard error was small for all thresholds, ranging from 2.13 for Amazonian mammals and birds to 8.17 for bats. Despite our results not agreeing with those reported in the original papers, likely due to differences in the regression models used and in the iterative fitting procedure to estimate model parameters, we opted to use our calculated threshold values since our method was consistent among studies.

Most piecewise regressions showed a trend similar to that observed in the original publications; an initial decline in diversity with forest cover, followed by a sudden decrease after a certain level of forest loss. However, the dataset on bats showed an increase in richness with increase forest loss until it reached a threshold. The same pattern can be observed in the original article. Lastly, the study on aquatic insects showed that beta-diversity decreases with increased forest cover, which increases when vegetation cover goes beyond the threshold. In summary, although our results showed some differences in trends along forest loss gradients, all showed clear thresholds.

Overall, there was no significant increase in heteroskedasticity with proximity to the threshold (χ² = 3.35, d.f. = 2, p = 0.19), suggesting it has limited use as a standard tool for forecasting biodiversity collapse due to forest loss. However, amphibians in the Atlantic Forest (Fig. 2d), and mammals and birds in the Amazon (Fig. 2g) did display evidence of increasing heteroskedasticity close to the threshold as we expected.

## Discussion

Threshold studies have previously been undertaken on a variety of taxa, responses, habitat types, landscape types and spatial scales^5,19–21,23^. The findings of these studies do not reach a clear consensus, and they are limited in number, restricting our ability to provide a general understanding of the mechanisms underlying the processes. Our findings provide only limited evidence that heteroskedasticity of biodiversity-related parameters can provide a general warning signal of impending threshold changes in communities along forest loss gradients. However, an apparent effect of increasing variance associated with threshold was evident for amphibians and for mammal and bird communities, suggesting that impending changes in some species assemblages might be predictable.

Amphibians, mammals and birds have been reported to be sensitive to landscape changes. Moreover, several species are known to be dependent on particular forest habitats^10,22–24^. However, their responses before a threshold have not been previously documented. We believe that increased heteroskedasticity near the threshold implied population instability. This instability might be a result of declining forest patch size and increased patch isolation. These changes in forest patches may have lasting effects on the population, exacerbating density dependent effects and increasing extinction risk for local populations. Furthermore, these changes may lead to decreased population sizes in fragments. If that were to occur, population size may also vary more within and among patches, potentially resulting in some local extinctions. These mechanisms could imprint variability in the alpha and beta diversity of some groups before a collapse^17^, such as we detected in the community composition of amphibians (occurrence data) in Atlantic Forest and the species richness for mammals and birds in the Amazon; both metrics whose sentitivity depends on the dynamic of local extinctions.

Our results have several implications for biodiversity conservation and monitoring in tropical forests. First, although the lack of replication within taxonomic groups prevent any generalization about context-dependent responses, our results do not support the idea that decreasing forest cover had a similar effect on different communities. Therefore, when proximity to a threshold is identified for a group, conservationists and managers should act rapidly to prevent the attainment of the extinction threshold in that specific group. This is a valid message even in cases where the mechanisms behind the warning signals are not clearly understood, because stopping forest loss around a threshold value is clearly valuable as a management practice.

Second, when dealing with multiple animal groups in an area, the focus should be given to the groups with the earliest threshold values. For example, aquatic insects reach community threshold responses at approximately 10% of forest loss while mammals do not display a similar response until near 60% (Fig. 2). So, one can use, for example, the response of aquatic insects as an early signal of biodiversity loss in response to the level of forest cover, even if there are no clear ecological relationships among different groups.

Third, thresholds of sensitive groups may be used to anticipate the response of other communities, though this method is yet to be proven as consistent among different geographic regions. For instance, an animal group may display different thresholds in different regions, thus being a useful indicator in one region where that threshold has been determined but not in another where it is yet to be examined. In this regard, we identified that Amazonian mammals and birds had much lower thresholds to forest loss than their Atlantic Forest counterparts (Fig. 2). This lower threshold could be related to the longer history of defaunation in the Atlantic Forest where even big forest patches are in fact largely empty of large mammals, such as jaguars, lowland tapirs, and white-lipped peccaries^25^. However, it still unclear if the sequence of threshold attainment by different animal groups is consistent across different regions. If this sequence is consistent, it would be a valuable and as yet untapped area of research on indicators of tropical forest loss. If proven to be true, for instance, the sequential thresholds among groups in the tropical forest could be caused by complex mechanisms linking them, such as species operating over a broad range of scales and cascade effects among dependent groups through ecological networks^26^.

Our study explored just one possible signal that could warn of an approaching threshold of community diversity. However, many other potential variables may exist including a direct measure of “critical slowing down” in a process, and transitioning between alternate states of a system^14,15^. Moreover, the sensitivity of different biodiversity metrics (e.g., richness, composition) as identifiers of thresholds is an almost unexplored topic. As our main focus here was to examine the practicality and ubiquity of heteroskedasticity as an indicator of a community warning signal, we explored the metrics already reported in the data of the underlying papers. However, it is important to consider two aspects. First, our results showed that the trends of some metrics along the gradient of forest loss vary according to the groups. For example, the richness of bats showed a positive response to native vegetation loss after the threshold while amphibians showed a gradual decline in richness. This finding suggests that solely evaluating a general trend of a metric may not adequately inform of a coming threshold. Second, the detectability and level of thresholds for different community metrics is a controversial issue. Although the majority of studies to date do not explore the sensitivity of different metrics, three cases can exemplify our point. Based on data from Atlantic Forest, Banks-Leite *et al*. documented that different community metrics for mammals, birds and amphibians showed similar patterns of thresholds^5^. However, in Amazonian streams, the threshold value was near 90% for compositional data (abundance) of aquatic insects, and below 20% (highly uncertain) for richness. Another study with aquatic insects in Brazilian savanna streams detected a threshold value around 50% for composition (abundance) but has failed to corroborate the threshold hypothesis using richness^27^. So, metrics that detected changes in the identity and abundance of species seem to be more sensitive to gradients of habitat loss than those based on richness which is more suceptible to compensatory mechanisms, such as substitution and invasions. Third, most studies on thresholds have focused on classic community metrics. We believe new avenues should be explored. For instance, different metrics from those used here, such as ecological network analysis or population parameters of sensitive species (e.g., movement behavior), could also be used to identify useful surrogates with which to detect thresholds and early signals of community collapse. Irrespective of the metric used, for threshold identification, research into the prediction of community thresholds will require substantial sampling, and a thorough understanding of the examined community. A particular challenge to this approach is that the availability of such data for the highly diverse and often under-studied tropical ecosystems along an entire gradient of forest loss is at present very limited.

Historically, most research on warning signals and thresholds have been focused on the temporal dimension. In our approach, we used a classic idea in ecology of considering a gradient in space to represent differences in time. These snapshots of biodiversity along a spatial gradient offer unique advantages for evaluating warning signals as they do not require intensive time series data which is rare in tropical community ecology^28^. However, neglecting the time since the habitat loss and fragmentation occurred could result in ineffectively identifying the thresholds and warning signals among different groups because extinctions follow deforestation may not occur immediately^29^. The fact that we detected warning signals for amphibians but not for mammals in the datasets from Atlantic Forest exemplifies this point. Considering that all habitat patches in this study were composed of a patchwork of secondary vegetation and that mammals and amphibians could differ in their extinction debt^27^, one would expect that the metrics of mammal and amphibian communities would show warning signals along a spatial gradient in different moments—a phenomenon which is extremely difficult to detect with snapshot survey approaches. In addition, most researchers argue that extinction debt is paid faster in extremely small size habitat remnants and is therefore a scale dependent phenomenon^30^. This finding calls for caution when comparing data based just on the percentage of remnant patches left in a landscape as this may mask functional effects in the remnant patches based on size and conditions. For example, we detected warning signals for mammals and birds in the Amazonian data which were collected in a natural landscape recently converted to pasture that still keep remnants of primary forest. Conversely, we did not find signals in the mammal community in data from the Atlantic forest, a region with a long history of land changes. In this way, we could imagine ghost (historic) warning signals experienced by the Atlantic Forest mammal community becoming no longer detectable. Thus, long-term biodiversity monitoring along entire gradients of forest loss at multiple scales may provide powerful insights into the spatial-temporal dynamics of thresholds and warning signals in tropical systems.

Sensitivity to changes, and the ability to detect system instability are critical aspects in determining a useful indicator in the context of the practical application of thresholds and warning signal information in forest monitoring. As positive data about thresholds is needed to test the prediction of warning signals and it is probable most studies in this area suffer from selective reporting^31^, using our analyses we could not go further in proposing community metrics for detecting thresholds without bias, though we may propose steps for selecting them. Considering that different metrics vary in their sensitivity to identify thresholds along forest loss gradients and, as we demonstrated here, not all variables that show thresholds also show a warning signal, we suggest a filtering process for selecting potential indicators of warning signals. First, one should evaluate the sensitivity of different community metrics (richness, beta diversity, so on) along a forest loss gradient and select those that respond clearly to the gradient. Second, potential thresholds along gradients should be evaluated for efficacy. Finally, one should evaluate the potential warning signals before approaching thresholds as we did or using other methods^32^. In summary, instead of looking for universal indicators, we believe that applying a filtering process is a more promising avenue for selecting effective warning signal indicators.

Despite future research challenges and a lack of clear, ubiquitous warning signals for all examined faunal guilds (which does not support the idea of a ubiquitous mechanism operating before threshold, such as the manifestation of critical slowing down), our findings support the identification of threshold responses as a potentially relevant future managerial tool for specific groups. While, the underlying mechanism is unlikely to be ubiquitous across numerous animal groups, our work supports the general message that while reducing habitat loss should remain a top priority for conservation planners^6^, the level of habitat loss at which communities can still be self-sustaining may be context and taxon specific.

## Methods

For each dataset we used, we recalculated diversity thresholds using a piece-wise regression^33,34^, following the procedure described by the authors in their original articles. In these regressions, the effect of deforestation on diversity was modelled, but we assumed there was a breakpoint in this relationship. This breakpoint represents a threshold in biodiversity, a point from which diversity decreases faster with deforestation. Piecewise regression estimates this point together with regression before and after it. We used the same measure of diversity that the authors used to detect these thresholds, as follows: composition of Amazonian aquatic insects^21^; richness of bats in Cerrado-Forest^20^; community composition of Amazonian mammals and birds^19^; community composition of Atlantic Forest mammals and bird^5^; community composition of Atlantic Forest (pooled groups)^5^; community composition of Atlantic Forest amphibians^5^; and community composition of Atlantic Forest birds^5^. “Community composition”, originally named “integrity” in Banks-Leite *et al*.^5^ is calculated as the Sorensen distance in species composition when comparing a community with no habitat fragmentation to that of the current community. All studies had vegetation cover as one of their independent variables, i.e. the percentage of the area that was not deforested. When comparing forest fragments with different levels of deforestation, all authors reported changes in their respective measures of diversity, with a clear threshold.

Once we recalculated the threshold values obtained by the original studies, we discarded data where deforestation levels were above the threshold since our interest was in measuring heteroskedasticy before the threshold. We then calculated the absolute standard residuals from the remaining data and pooled the absolute residuals from all models. Using standard residuals, guaranteed that the residuals will not be affected by the scale of the variable. Using absolute values implies that both negative and positive residuals represent heteroskedasticity. This pooled residuals variable was used as a reponse variable in the linear mixed model. In this model, standard residuals (residuals divided by their standard error) were the response variable, distance to threshold was a fixed independent variable and dataset was an independent random variable. To fit the model, we used a Nelder Mead optimization mechanism in the lme4 package in R^35,36^. To make inferences, we compared the previous model with a null model using an ANOVA. The null model was similar to the calculated model but did not contain distance to the threshold as a variable. If this model was significant, it indicates that residuals increase with proximity to threshold, and thus that heteroskedasticty increases with proximity to threshold.

We also examined each dataset separately, in addition to pooling them in the same model. For that, we again used the standard residuals obtained from a piecewise model. We then applied a linear regression for each dataset (Table 1). In all regressions, we used distance to the threshold as the independent variable. Residuals (as detailed below) were our target dependent variable; however, significance seems dependent on how residuals were transformed to a heteroskedasticty metric. Since we had no prior reason to prefer one transformation over another, we tested these using the three most common transformations: absolute residuals, squared residual (analogous to the Breusch–Pagan test for heteroskedasticity^37^) and ranked residuals (analogous to spearman correlation). In total, 21 tests were conducted (7 data sets x 3 independent variables).

**Table 1:** The effect of threshold proximity on variance of diversity metrics. According to our hypothesis, deforestation should increase flunctuations in diversity measures until it reaches a threshold at which large diversity loss occurs. We measured variance as the residuals in a segmented regression model with diversity as the dependent variable and deforestation as the independent variable. These residuals were then regressed with proximity to threshold, after three different transformations (absolute, squared and ranked). We rejected the null hypothesis if any of the three transformation was significant. Datasets: Am aquatic insects21; bats20; Am mammals and birds19; AF mammals5; AF pooled groups5; AF amphibians5; AF birds5. AF – Atlantic Forest; Am – Amazonian.

Dataset	Linear model on (absolute residuals)		Breusch–Pagan test (squared residuals)		Spearman correlation (ranked residuals)	
	F	P	Estimate	P	Rho	P
Am Aquatic insects	1.243	0.224	0.693	0.405	0.480	0.113
Bats	0.622	0.444	1.013	0.314	0.056	0.840
Am mammals & birds	4.234	**0.041**	2.838	0.092	−0.183	**0.028**
AF mammals	1.421	0.243	0.688	0.406	−0.288	0.121
AF pooled	0.261	0.612	0.085	0.770	−0.029	0.862
AF anurans	9.08	**0.005**	4.178	**0.040**	−0.488	**0.004**
AF birds	0.151	0.699	0.460	0.497	−0.059	0.730

### Data Availability

The datasets generated and/or analyzed during the current study are available from the corresponding author on request.
